# Presentation of Rare Phenotypes Associated with the *FKBP10* Gene

**DOI:** 10.3390/genes15060674

**Published:** 2024-05-23

**Authors:** Elena S. Merkuryeva, Tatiana V. Markova, Vladimir M. Kenis, Olga E. Agranovich, Ivan M. Dan, Yulia Y. Kotalevskaya, Olga A. Shchagina, Oxana P. Ryzhkova, Sergei S. Fomenko, Elena L. Dadali, Sergey I. Kutsev

**Affiliations:** 1 Research Centre for Medical Genetics, 115522 Moscow, Russia; 2 The Turner Scientific Research Institute for Children’s Orthopedics, 196603 Saint Petersburg, Russia; 3 The National Medical Research Center of Traumatology and Orthopedics Named after N.N. Priorov, 127299 Moscow, Russia; 4 Vladimirsky Moscow Regional Research and Clinical Institute, 61/2, Schepkina St., 129110 Moscow, Russia; 5 Research Institute of Medical Genetics, Tomsk National Research Medical Center of the Russian Academy of Sciences, 10, St. Ushaika River Embankment, 634050 Tomsk, Russia; 6 Charitable Foundation «BELA. Butterfly Children», Building 3, Room 1, Furmanny Lane, 105062 Moscow, Russia; 7 Genoanalytica Laboratory, 119234 Moscow, Russia

**Keywords:** osteogenesis imperfecta, Bruck syndrome, arthrogryposis, fractures, *FKBP10* gene

## Abstract

Pathogenic variants in the *FKBP10* gene lead to a spectrum of rare autosomal recessive phenotypes, including osteogenesis imperfecta (OI) Type XI, Bruck syndrome Type I (BS I), and the congenital arthrogryposis-like phenotype (AG), each with variable clinical manifestations that are crucial for diagnosis. This study analyzed the clinical-genetic characteristics of patients with these conditions, focusing on both known and newly identified *FKBP10* variants. We examined data from 15 patients, presenting symptoms of OI and joint contractures. Diagnostic methods included genealogical analysis, clinical assessments, radiography, whole exome sequencing, and direct automated Sanger sequencing. We diagnosed 15 patients with phenotypes due to biallelic *FKBP10* variants—4 with OI Type XI, 10 with BS I, and 1 with the AG-like phenotype—demonstrating polymorphism in disease severity. Ten pathogenic *FKBP10* variants were identified, including three novel ones, c.1373C>T (p.Pro458Leu), c.21del (p.Pro7fs), and c.831_832insCG (p.Gly278Argfs), and a recurrent variant, c.831dup (p.Gly278Argfs). Variant c.1490G>A (p.Trp497Ter) was found in two unrelated patients, causing OI XI in one and BS I in the other. Additionally, two unrelated patients with BS I and epidermolysis bullosa shared identical homozygous *FKBP10* and *KRT14* variants. This observation illustrates the diversity of *FKBP10*-related pathology and the importance of considering the full spectrum of phenotypes in clinical diagnostics.

## 1. Introduction

Currently, two primary phenotypes have been described that are associated with biallelic pathogenic variants in the *FKBP10* gene in the OMIM database [1]. These include osteogenesis imperfecta (OI) Type XI (MIM #610968) and Bruck syndrome Type I (BS I) (MIM #259450). According to the Human Gene Mutation Database (HGMD) [2], 63 nucleotide sequence variants have been identified that cause these phenotypes.

The *FKBP10* gene encodes FK506-binding protein 10 (FKBP65), which possesses peptidyl-prolyl isomerase (PPIase) activity, acting as a chaperone for lysyl hydroxylase-2 (LH2), thereby regulating the activity of LH2. A deficiency in bone-specific telopeptide LH2 results in the insufficient hydroxylation of lysine in the collagen telopeptide, leading to a reduction in hydroxylysyl pyridinoline cross-links and improper bone collagen cross-linking [3,4,5].

The molecular etiology of OI type XI was first established by Alanay et al. in 2010. By examining affected members of five related Turkish families with OI type XI, including multiple fractures, severe deformities of long bones, and progressive kyphoscoliosis in combination with epidermolysis bullosa simplex (EB), the authors identified homozygous variants in the *FKBP10* and *KRT14* genes that are located on the long arm of chromosome 17 and are inherited digenously, causing the occurrence of OI and EB in these families [6].

Subsequent studies by various authors have demonstrated that pathogenic variants in the *FKBP10* gene led to the emergence of another autosomal recessive disease, BS, characterized, in addition to multiple bone fractures, by congenital contractures of major joints [7,8,9,10,11]. In the modern classification structure of skeletal dysplasias, BS is included in the group of bone fragility disorders along with OI and is subdivided into Types I and II, associated with the *FKBP10* and *PLOD2* genes [12]. Additionally, in 2020, Essawi et al. described intrafamilial polymorphism of clinical signs in a large Palestinian family with ten affected members, manifesting signs of OI, BS, and only AG in one member, caused by a pathogenic homozygous variant in the *FKBP10* gene [13].

The variability of phenotypic manifestations of diseases associated with the *FKBP10* gene necessitates the study of the characteristics of their clinical presentations with different types and localization of pathogenic variants. Herein, we provide a detailed analysis of the clinical genetic characteristics of a patient sample with OI Type XI, BS I, and AG-like phenotype, caused by previously described and newly identified variants in the *FKBP10* gene.

## 2. Results

We observed 15 patients (9 males and 6 females) from 14 unrelated families presenting with clinical and radiological signs of OI (4/15) and BS (10/15), with 1 patient having the AG-like phenotype (1/15). In 1 of the 14 families, the disease was noted among biological siblings, while the other cases were isolated. In 5 families, the consanguinity of the probands’ parents was established. Additionally, two patients with Type I BS presented with simple EB from birth.

The clinical radiological characteristics of four patients with OI Type XI are presented in Table 1.

**Figure 1:**
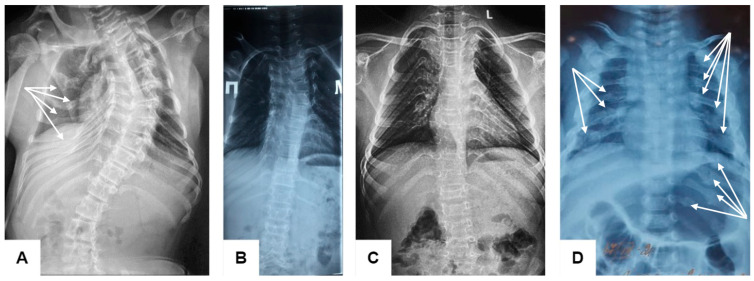
Radiographs of the thoracolumbar spine and chest in anterior views of patients with osteogenesis imperfecta Type XI of different ages ((**A**)—F1-P1, (**B**)—F2-P2, (**C**)—F2-P2, (**D**)—F4-P4): the chest is narrowed, especially in the upper segment; multiple rib fractures (white arrows); scoliosis of varying severity with a tendency to increase with age.

The study noted significant clinical variability among the four unrelated patients with OI Type XI, aged between 2.5 years and 21 years. The most severe course of the disease was observed in patients aged 21 years (F1-P1) and 2.5 years (F4-P4). The age of the first fracture among the examined patients varied from the intrauterine period (F4-P4) to 10 years (F2-P2). The total number of fractures was highest in the individual aged 21 years (F1-P1), and the frequency of fractures per year was higher in the child aged 2.5 years (F4-P4), despite both undergoing bisphosphonate therapy. All examined patients had scoliosis of varying severity, progressing with age. Interestingly, severe scoliosis in patient F1-P1 was accompanied by the preservation of vertebral body height (Figure 1).

The height of the proband F1-P1 was significantly below average (−7.02 SD), while the others were within normal limits or at the lower normal boundary. Among the three patients who underwent densitometry, a decrease in bone mineral density (BMD) was diagnosed. In the proband from the second family (F2-P2), who had not received treatment until the age of 10, severe osteoporosis was detected. In the assessment of extra-skeletal signs, a bluish sclera was found in two patients, but it was noted that this was particularly pronounced at an early age; only one patient was diagnosed with bilateral mixed hearing loss of the first degree from the age of 6, and none were found to have dentinogenesis imperfecta. Motor mobility was retained only in one 6-year-old patient (F3-P3), who could walk without assistance. A 10-year-old patient used crutches for mobility, and two others with a more severe course of OI (F1-P1, F4-P4) were unable to move independently.

**Figure 2:**
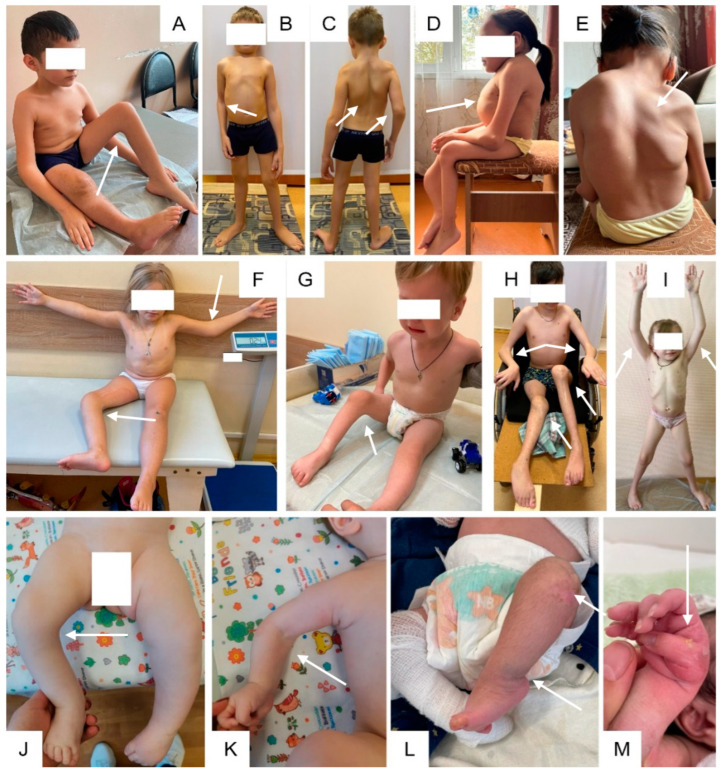
Clinical features of patients of different ages. Clinical features of patients of different ages with Bruck syndrome Type I. Bilateral or unilateral flexion contractures of joints (shown by arrows) (**A**,**B**,**F**–**K**). Scoliosis (**C**,**E**) and chest deformity (**D**). Note the epidermolysis bullosa simplex (**L**,**M**).

Detailed clinical and radiological data of 10 BS patients (7 boys and 3 girls) from 9 families are summarized in Table 2. Two patients with BS-I were combined with EB and grouped as BS-I/EB (Figure 2). Patients F6-P6 and F6-P7 are brothers from a monozygotic bi-amniotic twin pregnancy.

**Figure 3:**
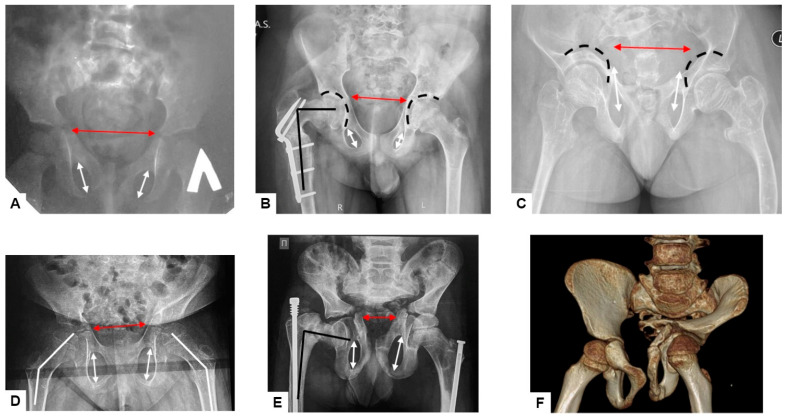
Anteroposterior radiographs of pelvis and hips from patients of the different ages: primary coxa valga (white lines) (**D**); secondary post-fracture coxa vara (black lines) (**B**,**E**); pelvic deformity—narrowing of the anterior part of the pelvic ring (red arrows) (**A**–**E**); protrusio acetabuli (black broken lines) (**B**,**C**); vertically oriented foramina obturatoriae (white arrows) (**A**–**E**). CT scan of the pelvis and hips (**F**).

**Figure 4:**
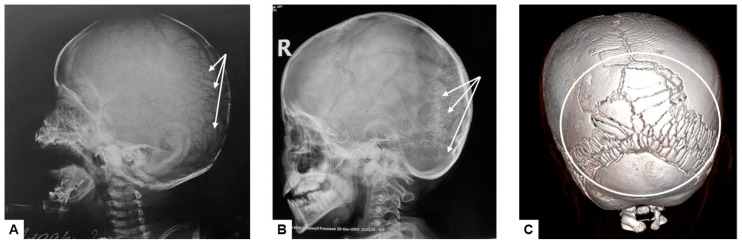
Abnormal ossification pattern of the skull vault: multiple wormian bones located in the region of lambdoid suture (white arrows on the lateral plane radiographs (**A**,**B**) and white circle on the reformed 3D CT image (**C**)).

The ages of the patients we examined ranged from 15 days old to 13 years old. All patients exhibited a common symptom of BS—joint contractures. Congenital contractures were present in 13 patients with BS (Figure 2). Flexion contractures variously affected the elbow, knee (bilateral or unilateral), and ankle joints. Wrist contractures were rare (2/13), and finger contractures were not observed in any of the patients. Fractures most often affected the femoral bones and were multiple, with four occurring intrauterinely. The highest number of fractures was observed in a 10-year-old patient (F8-P9), as he leads a socially active lifestyle, is able to move independently with crutches, and attends a regular school. Scoliosis of varying severity was detected in six patients. Deformation of the long tubular bones was noted in 7 out of 10 patients. The height of most examined patients (10/13) was low (ranging from −2.25 SD to −4.9 SD), due to progressive scoliosis, joint contractures, and deformation of long tubular bones. On pelvis X-rays, six out of eight patients showed protrusion of the acetabulum (either unilateral or bilateral) (Figure 3). This characteristic suggests that the bone was unusually soft and pliable as a result of low BMD, causing difficulties with mobility. It was noted that the progressive protrusion of the acetabulum in some patients led to severe motor impairments. Wormian bones were present on all available skull X-rays (Figure 4). Four patients showed platybasia on radiological examination of the skull, and one of them had basilar invagination. This same group of patients also exhibited a significant decrease in BMD (Z-score from −6.3 to −2.8), which possibly explains the development of such complications in them. Out of the 10 patients with BS reported here, 6 were treated with bisphosphonates, which does not allow for a comparison of the severity of osteoporosis in them over the course of the disease. All BS patients had mobility issues: 4 could move with crutches, 4 never had the ability to move independently, and 1 lost the ability to walk in the early years of life. It is worth noting that all examined patients had normal intelligence, and extra-skeletal signs such as blue sclera, imperfect dentinogenesis, and hearing loss were not found in any of the BS patients.

**Figure 5:**
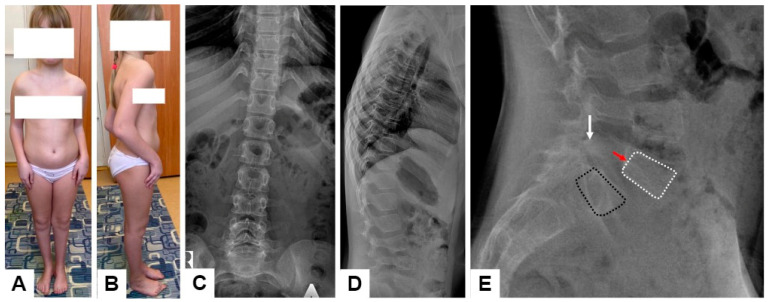
Appearance of the patient F14-P15 with AG-like phenotype (**A**,**B**). X-rays of the spine in frontal and lateral projections (**C**,**D**). Lateral radiographs of the lumbosacral region of the patient with spondylolytic spondylolisthesis: spondylolysis of the L5 vertebral arch (white arrows) and anterior displacement (red arrows) of the L5 bodies (white dotted lines) in relation to the S1 (black dotted lines) (**E**).

It is interesting to note the clinical features of an 11-year-old patient, characterized only by contractures without fractures (F14-P15) (Figure 5). At birth, she was found to have bilateral clubfoot and flexion contractures of the elbow joints, for which the Ponseti method (casting and Achilles tenotomy), arm casting (bar with hinges), and reconstructive foot surgeries at ages 3 and 5 were applied. To date, the girl has not had any fractures. Upon examination, the patient’s height was −0.72 SD, body weight −0.11 SD, and head circumference 0.32 SD, and flexion contractures of the elbow joints was as follows: right extension to 110 degrees, left to 100, and flexion to 40 degrees (active and passive). The hands were in palmar flexion position, actively corrected to dorsal extension. The right foot was in a neutral position, the left in equino–cavus–varus. Radiological features included positional thoracolumbar kyphosis with normal height and shape of the vertebral bodies and intervertebral disc (Figure 5C,D). Asymptomatic spondylolytic spondylolisthesis of L5 was revealed on the lateral radiographs (Figure 5E).

As a result of molecular-genetic analysis, 10 different variants in the *FKBP10* gene were identified (Table 3).

The spectrum of 10 identified FKBP10 variants consists of frameshift variants: 4, missense: 3 (one of which affects splicing), splice-site, nonsense, and deletion: one variant each. Three *FKBP10* variants were identified for the first time in patients with BS-I, c.1373C>T (p.Pro458Leu), c.21del (p.Pro7fs), and c.831_832insCG (p.Gly278Argfs), occurring in a compound heterozygous state with previously described variants. Sanger sequencing validation and segregation in families confirmed their presence in patients in the trans position. According to the American College of Medical Genetics (ACMG) pathogenicity criteria, the newly identified variants were classified as pathogenic (Table 3).

In this study, a recurrent variant c.831dup (p.Gly278Argfs) was registered in five patients with *FKBP10*, accounting for 17.9% of the alleles in the examined sample. In the literature, this variant in a homozygous state was first registered in three Mexican siblings with OI XI [6]. In 2011, Kelley et al. identified the same variant in a homozygous state in two siblings with a moderately severe form of OI XI; however, only one of them had congenital joint contractures, and it was also found in another patient with BS [8]. In our study, this variant (c.831dup) in a homozygous state was found in a patient with BS-I (F5-P5) and in siblings with BS-I (F6-P6,7) in a compound heterozygous state with the newly identified variant c.1373C>T (p.Pro458Leu), and in two patients with OI XI in a compound heterozygous state with previously described variants.

Additionally, four more patients in our study were found to have the variant c.344G>A (p.Arg115Gln) in the *FKBP10* gene, which was registered in 14.3% of the alleles of the sample. According to the literature, this variant was more commonly found in a homozygous state in patients both with OI XI and BS-I and was described in a compound heterozygous state with c.831dup (p.Gly278Argfs) in a patient with BS-I [8,10,14]. In the current study, this variant was registered in a patient (F8-P9) with the BS-I type in a compound heterozygous state with the newly identified variant c.21del (p.Pro7fs) and in two more patients with BS-I type, with previously described variants c.976del (F7-P8), c.1256G>A (F11-P12), as well as in a compound heterozygous state with c.1016G>A in one patient with the AG-like phenotype (F16-P18).

Two unrelated patients, ethnic Tuvans with different phenotypes of OI XI (F4-P4) and BS-I (F9-P10), were found to have an identical homozygous variant c.1490G>A (p.Trp497Ter), previously described in patients with only OI XI [14].

Furthermore, two unrelated patients (F12-P13 and F13-P14) with BS combined with congenital simple EB were found to have a homozygous deletion of 33 base pairs in the reading frame (c.321_353del), leading to the deletion of 11 amino acids (p.Met107_Leu117del) in the *FKBP10* gene and a homozygous variant c.612T>A (p.Tyr204Ter) in the *KRT14* gene. These same homozygous variants had been previously identified in patients from the northern part of Turkey [6,15].

## 3. Discussion

To date, the diversity of phenotypes associated with the *FKBP10* gene is represented by autosomal recessive forms that constitute a continuum: from bone fragility (OI Type XI) and its combination with congenital joint contractures (BS I), to possible cases of AG without fractures [13]. Clear geno-phenotypic correlations for these diseases associated with the *FKBP10* gene have not been established. Furthermore, seven variants in this gene, in a homozygous state, can lead to either BS I or OI XI in patients [2].

**Figure 6:**
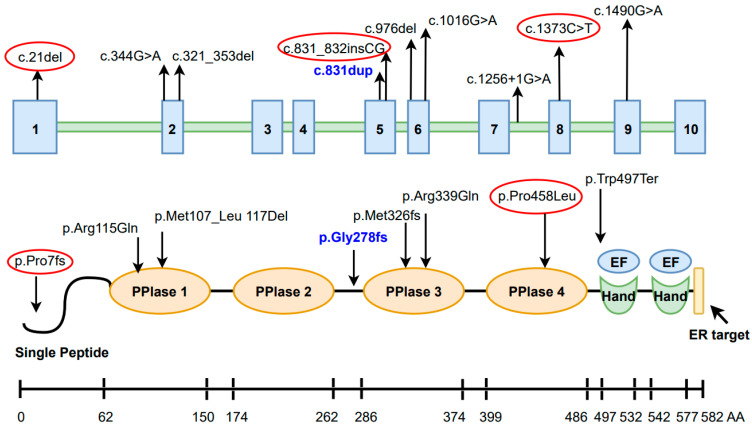
Schematic representation of the *FKBP10* gene and FKBP65 protein. Upward arrows indicate variants identified in the *FKBP10* gene, and downward arrows indicate the location in various domains of the FKBP65 protein in this study. Variants not previously described are outlined in red. The recurrent variant is highlighted in blue.

The protein product of the *FKBP10* gene is FK506-binding protein 10 (FKBP65), which possesses peptidyl-prolyl isomerase (PPIase) activity. FKBP65 features four PPIase domains, catalyzing the interconversion of cis/trans isomers of peptidyl-prolyl bonds in proteins containing proline; a signal peptide; two EF/Hand domains, and a presumed endoplasmic reticulum retention sequence (Figure 6). Since approximately one-sixth of collagen’s sequence consists of proline residues, this conversion is a limiting step in the folding process of emerging proteins [16]. FKBP65 prevents premature cross-link formation between type I procollagen chains and facilitates accurate registration and folding into a triple helix of collagen with subsequent transport [17,18]. Moreover, FKBP65 can indirectly modulate the cross-linking of type I collagen and is crucial for the stability or activity of LH2 [10,17]. It is known that inactivating mutations in *FKBP10* can lead to the loss of FKBP65 function, a noticeable decrease in lysyl hydroxylation, and unstable secreted type I procollagen [8,19,20].

In the patients from the 14 families we examined, 10 pathogenic variants in the *FKBP10* gene were identified, of which 2/3 were nonsense or led to a frameshift. According to the literature, pathogenic *FKBP10* variants are distributed throughout the gene but are most commonly located in exons 5 and 6. In our study, pathogenic variants located in these exons accounted for 53.6% of the patient sample (Figure 6).

In the current study, the recurrent variant in the *FKBP10* gene, c.831dup (p.Gly278Argfs), was identified in five patients, accounting for 17.9% of the alleles in the examined sample. This variant in exon 5 of the *FKBP10* gene reflects slippage during replication in a sequence containing seven consecutive cytosines and has been repeatedly described in patients with OI and BS-I from various countries [6,7,8,10,15,21,22]. Our own results and those previously conducted suggest that codon 831 of the *FKBP10* gene may represent a “hotspot”.

As a result of this study, three new variants in the *FKBP10* gene were identified: c.1373C>T (p.Pro458Leu), c.21del (p.Pro7fs), and c.831_832insCG (p.Gly278Argfs). They were found in a compound heterozygous state with previously described variants and were clinically associated with the BS I type.

The new variant c.1373C>T (p.Pro458Leu) in the *FKBP10* gene was registered in a compound heterozygous state with the recurrent variant c.831dup in twin siblings (F6-P6, 7). One of them (F6-P6) had bilateral clubfoot at birth, for which the Ponseti method was applied, as well as flexion contractures of both elbow joints and the right knee joint. In the twin brother (F6-P7), unilateral flexion contractures of the right elbow and right knee joints were diagnosed at birth. Both children had registered rib fractures at birth, and both underwent surgical treatment for unilateral inguinal hernia and stepwise casting of joint contractures. By the age of 2, they were unable to walk independently.

Another new variant in the *FKBP10* gene c.21del (p.Pro7fs) in a compound heterozygous state with the substitution c.344G>A (p.Arg115Gln) was identified in a patient with BS I (F8-P9). The identified single-nucleotide deletion c.21del is located in the first exon of the *FKBP10* gene and leads to a frameshift starting from codon 7. Previously, a duplication at the same position, c.21dup p.(Ser8Glnfs*67) in a homozygous state, was registered in a patient from a consanguineous family from Egypt with a moderately severe course of OI [21]. According to predictions by VEP (Ensemble’s Variant Effect Predictor), these variants might be NMD-escaping, but this requires confirmation by functional studies. The severity of BS I in the patient we examined (F8-P9) was due to congenital contractures and rib fractures. The child was able to walk independently from the age of 3 years, but from this period, the frequency of fractures sharply increased. It is known from the history that the patient’s sister with a severe course of BS died at the age of 4 years as a result of an injury.

In this study, another variant in the *FKBP10* gene, c.831_832insCG (p.Gly278Argfs), was registered for the first time in a compound heterozygous state with another variant c.976del (p.Met326fs) in a patient (F10-P11) with congenital clubfoot and rib fractures at birth. The variant c.976del (p.Met326fs) in a homozygous state was previously described in a patient from a related Iranian family with mild joint contractures, kyphoscoliosis, muscle atrophy, progressive deformation, and multiple bone fractures [23]. The frequently occurring insertion of a single-nucleotide c.831dup, as well as our newly found two-nucleotide insertion c.831_832insCG, lead to a frameshift starting from codon 278 and a translational termination (p.Gly278Argfs) 17 amino acids lower, in the third PPIase domain of the protein. Patient F10-P11 did not receive bisphosphonate treatment until the age of 7. Upon examination, low growth and inability to move independently was noted. Pelvic X-rays confirmed multiplanar pelvic deformation with narrowing due to the left half. The MRI of the brain revealed MRI signs of basilar impression and Arnold-Chiari II malformation.

The second most common variant in the study presented was the replacement c.344G>A (p.Arg115Gln) in a compound heterozygous state with other variants in three patients with BS-1 and one with the AG-like phenotype. Previously, this variant in a homozygous state was discovered by Schwarze et al. in 2013 in patients with BS from Saudi Arabia, while Umair et al. in 2016 found it in three affected members from a consanguineous Pakistani family with a severe course of OI [10,14]. Interestingly, in the current study, the variant c.344G>A in a compound heterozygous state was found with the variant c.1016G>A in patient F16-P18, who has been observed since birth with a diagnosis of AG and underwent surgical treatment for contractures. By the age of 10, she had not experienced fractures of the long tubular bones or the spine, and there was no osteoporotic structure of the bone tissue on radiological examination. Previously, a functional analysis was conducted for the variant c.1016G>A in the *FKBP10* gene, which showed that it affects splicing and leads to the skipping of exon 6 [24]. To our knowledge, there is a description of a single six-year-old patient with AG associated with a homozygous variant c.391+4A>T in the second intron of the *FKBP10* gene [13]. The authors described a broad intrafamilial polymorphism of clinical signs, manifesting as signs of OI, BS, and AG among the affected members.

In this research, a case of OI Type XI in one patient (F4-P4) and BS-I in another (F9-P10), both unrelated and of Tuvinian ethnicity, was caused by the nonsense variant c.1490G>A (p.Trp497Ter) in a homozygous state. This variant, in a homozygous state, has been previously described in three patients from a consanguineous Pakistani family with a severe course of OI Type XI [14]. The presence of significant clinical polymorphism in such cases allows for the conclusion that the genetic background and epigenetic modifiers might influence the phenotype of the disease, which remains to be determined. Additionally, it is noteworthy that in our examined patient F4-P4, signs of nephrocalcinosis were detected by ultrasound from the age of one year, and one patient at the age of ten in the study by Umair et al. was observed to have kidney stone disease.

Our study noted two cases of children born with a combination of BS-I and EB in consanguineous Tajik families (F12-P13 and F13-P14). Previously, similar phenotypes were identified in patients from the northern part of Turkey and convincingly demonstrated a founder effect [6,15]. Considering these data, a search for previously registered homozygous variants in the *FKBP10* gene c.321_353del (p.Met107_Leu117del) and *KRT14* (c.612T>A (p.Tyr204Ter) was conducted by direct Sanger sequencing in our examined patients, which confirmed the presence of these variants.

## 4. Materials and Methods

The study material consisted of comprehensive examination data from 15 patients from 14 unrelated families, ranging in age from 15 days to 21 years, presenting with clinical and radiological manifestations of OI, BS, and the AG-like phenotype. The following methods were employed for diagnostic refinement: genealogical analysis, clinical examination, neurological assessment using standard techniques with evaluation of the psycho-emotional sphere, X-ray examination, CT, densitometry, whole exome sequencing, and direct automated Sanger sequencing.

Whole exome sequencing: Genomic DNA was extracted from peripheral blood leukocytes using the QIAamp DNA Blood Mini Kit (Qiagen, Hilden, Germany) according to the manufacturer’s protocol. The concentration of DNA, fragmented DNA after ultrasonic treatment, libraries, and the final pool were measured on a Qubit 2.0 device using manufacturer’s reagents (Qubit BR, Qubit HS, Life Technologies Corporation, Eugene, OR, USA) according to the standard protocol. The representation of DNA fragments of various lengths after ultrasonic treatment, libraries, and the final pool were monitored on a TapeStation 4200 (Agilent Technologies, Waldbronn, Germany) device using the manufacturer’s reagents (high sensitivity DNA D1000) according to the standard protocol. For sample preparation, a methodology of selective capture of DNA regions corresponding to the coding areas of 19,396 genes (IlluminaTruSeq^®^ Exome Kit and IDT xGen^®^ Exome Research Panel set, San Diego, CA, USA) was used. The average coverage of the patient’s whole exome was over ×60; the percentage of target areas with coverage ≥ ×10 was 99%; and the uniformity of coverage (uniformity Pct > 0.2 * mean) was 99%. Primary sequencing data processing was performed using the standard automated algorithm provided by Illumina for data analysis, available at https://basespace.illumina.com (accessed on 12 June 2023).

Validation of variants identified by massive parallel sequencing in the probands, as well as genotyping of siblings and parents, was carried out by direct automated Sanger sequencing according to the manufacturer’s protocol on an ABIPrism 3500xl device (Applied Biosystems, Waltham, MA, USA). Primer sequences were selected according to the reference sequence of the target regions of the *FKBP10* gene (NM_021939.4) and *KRT14* (NM_000526.5) genes.

All patients involved in the study provided written informed consent for the clinical examination and the publication of their anonymized data. The study was conducted in accordance with the Declaration of Helsinki and approved by the Institutional Review Board of the Research Center for Medical Genetics, Russia (protocol code: 10/1, Date: 8 November 2021).

## 5. Conclusions

The analysis of the clinical genetic characteristics of the examined patient sample confirms the diversity of *FKBP10*-related pathology and emphasizes the importance of considering the entire range of phenotypes in clinical diagnosis, ranging from fractures without contractures and fractures with contractures to the presence of only congenital contractures. The results of the studies expand the spectrum of new nucleotide variants in the *FKBP10* gene. Clear genotype–phenotype correlations for OI, BS, and AG associated with the *FKBP10* gene could not be established. The possibility of developing both OI Type XI and BS-I in patients with the variant c.1490G>A (p.Trp497Ter) has been shown. The study’s results convincingly support the existence of a “hotspot” in codon 831 of the *FKBP10* gene. When combining EB with congenital contractures or OI, diagnosis can be narrowed down to targeted search for one variant in each of the *FKBP10* and *KRT14* genes, which significantly simplifies and reduces the cost of their molecular-genetic diagnosis. In other cases, whole exome sequencing should be used to refine the genetic variant of the nosological form.

## Figures and Tables

**Table 1:** Clinical features of patients with autosomal recessive osteogenesis imperfecta type XI.

Patient	F1-P1	F2-P2	F3-P3	F4-P4
Parental consanguinity	+	−	−	−
Ethnicity	Armenians	Russian	Russian	Tuvans
Age, year	21	10	6	2.5
Sex, M/F	F	M	F	M
Age of 1st fracture, year ^1^	0.75	10.0	5.25	i/f, at birth
Fracture site ^2^	2, 3, 4, 5, 6	5	5, 7	1, 2, 4, 5, 6
Fractures total (n)	60	2	3	10
Number of fractures/year	2.85	0.18	0.41	4
Scoliosis (degree)	IV	II	I	I
Height, (SD Score)	−7.02	−0.93	−1.26	1.8
BMD ^3^ (L1–L4) (g/cm^2^)	0.549	0.195	0.434	−
Z-score (L1–L4)	−1.5	−5.5	−2.1	−
Blue sclera (+/−)	+	−	+	−
Dentinogenesis imperfecta (+/−)	−	−	−	−
Hearing loss (+/−)	+	−	−	−
Mobility (+/−)	− (wheelchair)	+ (crutches)	+	−
Age of treatment initiation by BP ^4^, year	5	10	6	0.16

^1^ I/f, Intrauterine fracture. ^2^ The fracture sites are 1 = skull, 2 = rib, 3 = humerus, 4 = ulna and radius, 5 = femur, 6 = tibia and fibula, 7 = vertebra. ^3^ BMD, bone marrow density. ^4^ BP, bisphosphonates.

**Table 2:** Clinical and radiological characteristics of the studied patients with Bruck syndrome.

Patient		F5-P5	F6-P6	F6-P7	F7-P8	F8-P9	F9-P10	F10-P11	F11-P12	F12-P13	F13-P14
Phenotype		BS-I	BS-I	BS-I	BS-I	BS-I	BS-I	BS-I	BS-I	BS-I/EB	BS-I/EB
Parental consanguinity		+	−	−	−	−	−	−	−	+	+
Ethnicity		Uzbeks	Russian	Russian	Russian	Russian	Tuvans	Ukrainian	Russian	Tajiks	Tajiks
Age, year		13	2	2	8	10	10	7	13	0.08	5
Sex, M/F		M	M	M	F	M	F	F	M	M	M
Congenital contracture		+	+	+	+	+	+	+	+	+	+
Talipes equinovarus		−	+	−	rt+	+	−	+	+	+	+
Knee ^1^		+	rt+	rt+	rt+	lt+	−	−	+	+	+
Elbow ^1^		−	+	rt+	lt+	rt+	+	−	+	+	+
Wrist		−	−	−	−	−	−	−	+	+	−
Bullous lesion		−	−	−	−	−	−	−	−	+	+
1st fracture, year ^2^		2	i/f	i/f	0.16	i/f	1.6	at birth	at birth	−	i/f
Fracture	1–18 ys ^3^	+	+	+	+	+	−	+	+	−	+
	0–12 m ^3^	+	−	−	+	+	+	+	+		+
Fracture site ^4^		3, 4, 5, 6	2	2	5, 6	2, 3, 4, 5, 6	5, 6	2, 3, 5, 6	2, 5, 6		2, 5, 6
Fractures total (n) ^5^		20	2	2	10	>50	20	7	20		mpl
Scoliosis (degree)		III	−	−	II	I	II	−	II	−	II
Long bone deformities (UL, LL) ^6^		LL	LL	LL	LL	UL	LL	−	−	−	LL
Acetabular protrusion ^7^		+	−	−	ND	+	+	+	+	ND	+
Wormian bones ^7^		+	+	+	ND	ND	+	+	ND	ND	ND
Platybasia ^7^		+	−	−	ND	ND	+	+	+	ND	ND
Height, (SD Score)		−4.9	−0.35	−0.35	−3.08	−2.98	−4.55	−2.64	−2.25	0.10	−2.91
BMD (L1–L4) (g/cm^2^)		0.362	−	−	0.843	0.543	0.390	0.394	0.588	−	−
Z-score (L1–L4)		−6.3	−	−	+0.1	0.7	−3.6	−3.4	−2.8	−	−
Mobility		−	−	−	+ (crutches)	+ (crutches)	−	+ (crutches)	−		+ (crutches)
Never able to walk		+	+	+			+				
Lost the ability to walk									+		
Age of treatment initiation by BP ^8^, year		13	−	−	2	4	5	−	7	−	0.16

^1^ Rt, right; Lt, left. ^2^ I/f, Intrauterine fracture. ^3^ M, months; ys, years. EB, epidermolysis bullosa. ^4^ The fracture sites are 2 = rib, 3 = humerus, 4 = ulna and radius, 5 = femur, 6 = tibia and fibula, 7 = vertebra. ^5^ mpl, multiple. ^6^ UL, upper limb; LL, lower limb. ^7^ ND, no data. ^8^ BP, bisphosphonates.

**Table 3:** *FKBP10*/*KRT14* variants spectrum of the patients.

Patient	Clinical Diagnosis	Gene	DNA Change	Protein Change	Ex.	Literature/ ACMG Criteria
F1-P1	OI-type XI	*FKBP10*	c.831dup	p.Gly278Argfs	5	+
			c.1256+1G>A		7i	+
F2-P2	OI-type XI	*FKBP10*	c.1016G>A	p.Arg339Gln	6	+
			c.1256+1G>A		7i	+
F3-P3	OI-type XI	*FKBP10*	c.831dup	p.Gly278Argfs	5	+
			c.1016G>A	p.Arg339Gln	6	+
F4-P4	OI-type XI	*FKBP10*	c.1490G>A homozygote	p.Trp497Ter	9	+
F5-P5	BS-I	*FKBP10*	c.831dup homozygote	p.Gly278Argfs	5	+
F6-P6, 7	BS-I	*FKBP10*	c.831dup	p.Gly278Argfs	5	+
			c.1373C>T	p.Pro458Leu	8	Novel Pathogenic (PM2, PP3, PM3, PP4)
F7-P8	BS-I	*FKBP10*	c.344G>A	p.Arg115Gln	2	+
			c.976del	p.Met326fs	6	+
F8-P9	BS-I	*FKBP10*	c.21del	p.Pro7fs		Novel Pathogenic (PM2, PVS1, PM3)
			c.344G>A	p.Arg115Gln	2	+
F9-P10	BS-I	*FKBP10*	c.1490G>A homozygote	p.Trp497Ter	9	+
F10-P11	BS-I	*FKBP10*	c.976del	p.Met326fs	6	+
			c.831_832insCG	p.Gly278Argfs	5	Novel Pathogenic (PM2, PVS1, PM3)
F11-P12	BS-I	*FKBP10*	c.344G>A	p.Arg115Gln	2	+
			c.1256+1G>A		7i	+
F12-P13	BS-I/EB	*FKBP10*	c.321_353del homozygote	p.Met107_Leu117del	5	+
		*KRT14*	c.612T>A homozygote	p.Tyr204Ter	3	+
F13-P14	BS-I/EB	*FKBP10*	c.321_353del homozygote	p.Met107_Leu117del	5	+
		*KRT14*	c.612T>A homozygote	p.Tyr204Ter	3	+
F14-P15	AG	*FKBP10*	c.344G>A	p.Arg115Gln	2	+
			c.1016G>A	p.Arg339Gln	6	+

Ex.—exon, OI—osteogenesis imperfecta, BS—Bruck syndrome, EB—epidermolysis bullosa, AG—arthrogryposis.

## Data Availability

Data are contained within the article.
